# Dataset on cellulose nanoparticles from blue agave bagasse and blue agave leaves

**DOI:** 10.1016/j.dib.2018.03.028

**Published:** 2018-03-10

**Authors:** Eduardo Robles, Javier Fernández-Rodríguez, Ananda M. Barbosa, Oihana Gordobil, Neftali L.V. Carreño, Jalel Labidi

**Affiliations:** a Biorefinery Processes Research Group, Chemical & Environmental Engineering Department, University of the Basque Country UPV/EHU, Plaza Europa 1, 20018 Donostia, Spain; b Materials Science and Engineering, Technology Development Center, Federal University of Pelotas, Gomes Carneiro 1, 96010610 Pelotas, RS, Brazil

## Abstract

These data and analyses support the research article “Production of cellulose nanoparticles from blue agave waste treated with environmentally friendly processes” Robles et al. [1]. The data and analyses presented here include fitted curves for selected carbons of the ^13^C CP-MAS NMR analysis; SEM images of the raw and bleached fibers, graphics with chemical composition and visual images of the fibers throughout the process.

**Specifications table**

**Table:** 

Subject area	*Chemistry*
More specific subject area	*Cellulose, nanocellulose*
Type of data	*Figures and graphs*
How data was acquired	*SEM (JSM-6400 F Scanning electron microscope, JEOL)*
	*NMR (AVANCE-500 Digital NMR spectrometer, Bruker)*
	*AFM (Multimode TM-AFM with NanoScope IIIa controller, Bruker)*
	*Images (COOLPIX S6400, Nikon)*
Data format	*Raw micrographs, fitted curves, analyzed graphics.*
Experimental factors	*SEM samples coated with graphite.*
	*AFM samples coated with*
Experimental features	*NMR data were recorded in solid state with Cross Polarization/Magic Angle Spinning*
Data source location	*AFM and visual images were taken at the Faculty of Engineering, Gipuzkoa, NMR data were collected at the Joxe Mari Korta Center, both within the Campus of Gipuzkoa of the University of the Basque Country UPV/EHU SEM images were recorded at the Faculty of Science and Technology of the University of the Basque Country UPV/EHU in the Campus of Biscay*
Data accessibility	*Data is accessible in the present document.*
Related research article	*Production of cellulose* nanoparticles *from blue agave waste treated with environmentally friendly processes.*

**Value of the Data**

• These data provide the micrographics, chemical composition and crystallinity data of CNC and CNF from blue agave waste.
• These data provide further information about NMR analyses of the different cellulose nanoentities.
• These data allow researchers to extend the comprehension of the related article.

## 1 Data

**Fig. 1:**
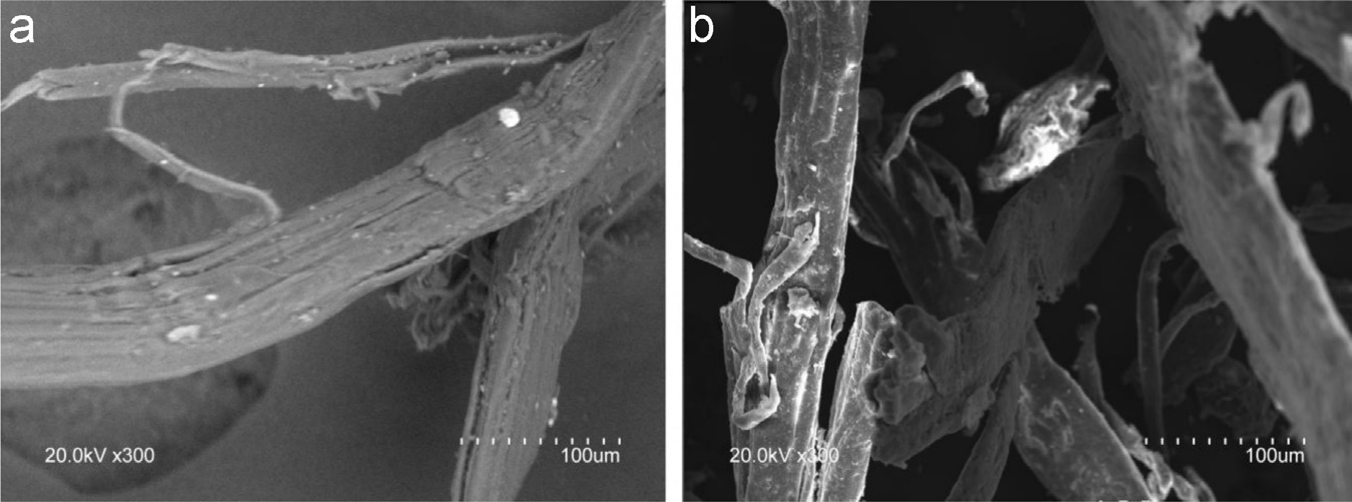
SEM images of a) blue agave leaf fibers and b) blue agave bagasse fibers as received.

**Fig. 2:**
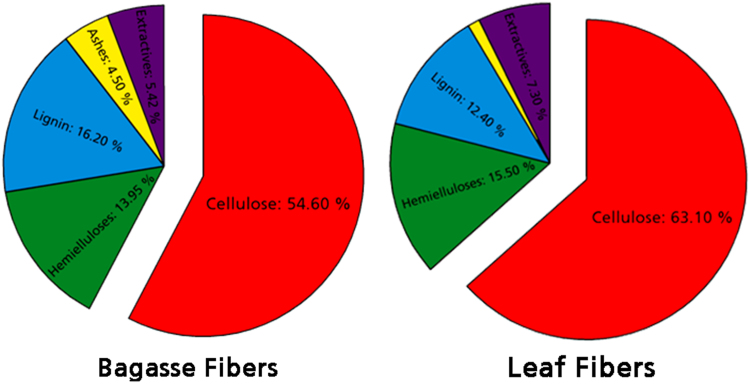
Chemical composition of leaf and bagasse fibers as obtained from TAPPI standard methods.

**Fig. 3:**
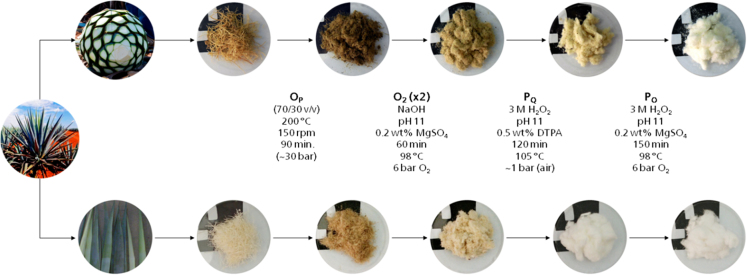
Schematic depiction of the fibers after each treatment.

**Fig. 4:**
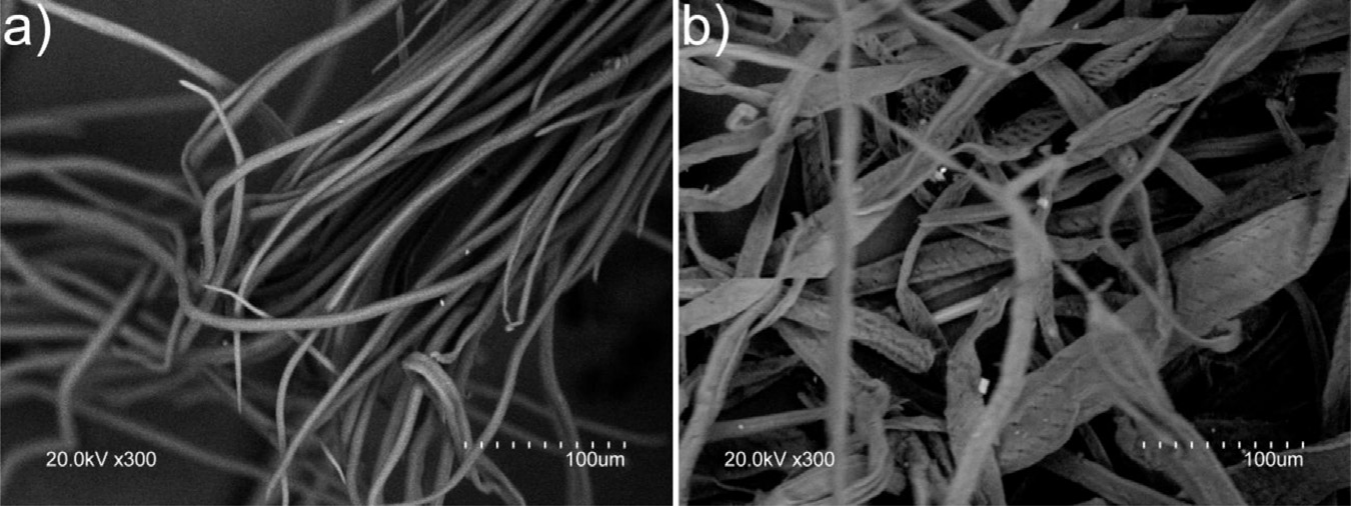
SEM images of a) blue agave leaf fibers and b) blue agave bagasse fibers after Organosolv pulping and TCF bleaching.

**Fig. 5:**
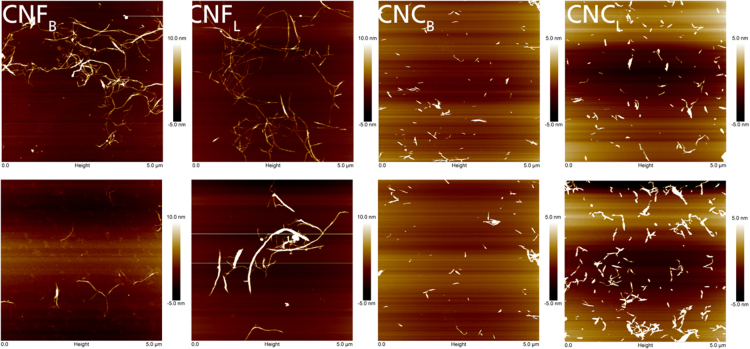
AFM images of CNF (left) normalized height from −5 to 10 nm and CNC (right) normalized height from −5 to 5 nm.

**Fig. 6:**
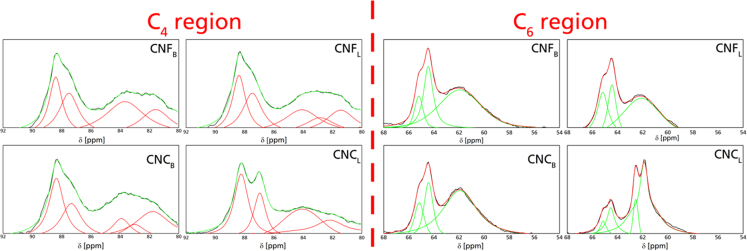
Fitted curves for the C_4_ and C_6_ regions as obtained by ^13^C NMR.

The data in this article contains information on the chemical composition (Fig. 2), visual aspect of the fibers through pulping and bleaching (Fig. 3), SEM (Fig. 1 and Fig. 4) and AFM (Fig. 5) micrographics as well as NMR (Fig. 6) analysis of different cellulose nanoentities obtained from blue agave (A*gave tequilana* Weber *var. azul*) waste. For more information, please refer to Robles et al. [1].

## 2 Materials and methods

SEM images were obtained with a Scanning electron microscope Hitachi S-3400N with field emission cathode, with a lateral resolution of 10–11 Å at 20 kV.

Chemical characterization was done according to standard methods [2], [3], [4], [5], [6].

13C NMR spectrometry was performed at a frequency of 250 MHz with an acquisition time of 0.011 s, at room temperature. The spectrum was recorded over 32 scans and water was used as solvent for all the nanocelluloses.

CrystallinityCrystallinity indexes were calculated as follows:

Segal Index [7]:

$${\mathrm{Cr}.I.}_{\mathrm{Segal}}=\;100\;\times \;\frac{I_{200}-I_{\mathrm{AM}}}{I_{\mathrm{tot}}}$$

In which *I*_200_ corresponds to the main crystalline domain at around 23°, and *I*_AM_ is the scatter of the amorphous cellulose, which has its highest intensity around 2*θ* = 18°.

Peak fitting:

(1) $${\mathrm{Cr}.I.}_{\mathrm{Peak fitting}}=\;100\;\times \;\frac{\int_{{2\theta }_{1}}^{{2\theta }_{2}}S_{11®0}d2\theta +\int_{{2\theta }_{1}}^{{2\theta }_{2}}S_{110}d2\theta +\int_{{2\theta }_{1}}^{{2\theta }_{2}}S_{200}d2\theta +\int_{{2\theta }_{1}}^{{2\theta }_{2}}S_{004}d2\theta }{\int_{d2\theta }^{d2\theta }S_{\mathrm{tot}}d2\theta }$$

In which the sum of the areas correspondent to the diffraction of crystalline planes is assumed to be the area of the crystalline region, being 2*θ*_1_ and 2*θ*_2_ the limits of the fitted signal for the corresponding crystalline domains (*S*_1–10,_ *S*_110,_ *S*_200,_ *S*_004_); while *S*_tot_ corresponds to the total area [8], [9], [10]. Least square iterations were done until coefficient of determination *R*^2^ ≥ 0.997 was achieved, which corresponds to a 99.7% accurate fitting.

C4-NMR:

(2) $${\mathrm{Cr}.I.}_{\mathrm{NMR}}=\;100\;\times \;\frac{\int_{87}^{93}\mathrm{Scrys dx}}{\int_{80}^{93}\mathrm{Stot dx}}$$

In which Scrys corresponds to the crystalline region of the C4 spectra (from 87 to 93 ppm) while Stot corresponds to the total area of the C4 region which includes crystalline and amorphous contribution.

Crystallite domain sizes (*δ_hkl_*) were estimated with the Scherrer equation [11], [12]. using the peaks corresponding to the crystalline regions:

$$d_{\mathrm{hkl}}=\frac{\kappa \lambda }{H_{\mathrm{hkl}}\cos\theta }$$

The different crystallinities, as well as the contributions of each crystallite domain size, is present in Table 1

**Table 1:** Solid-state properties of the different nanoparticles as obtained from XRD and NMR. Cr.I_X_ corresponds to the crystallinity index calculated with: SI-Segal Index, PF-Peak fitting, C_4_-NMR C_6_ NMR region; *δ_hkl_* is the crystallite size approximated with the Scherrer equation.

Sample/Method	Cr.I_SI_ [%]	Cr.I_PF_ [%]	Cr.I_C4_	*δ*1_1-10_ [Å]	*δ*_110_ [Å]	*δ*_200_ [Å]
CNF_B_	75.89	73.75	50.50	60.60	55.61	59.97
CNF_L_	72.29	72.16	51.32	38.15	30.03	47.44
CNC_B_	84.68	78.12	52.01	71.22	71.40	71.94
CNC_L_	87.10	82.65	63.76	49.85	37.47	44.83
