# Supplementary material for: Dataset on cellulose nanoparticles from blue agave bagasse and blue agave leaves
Source: Data Brief. 2018 Mar 10;18:150–5. doi: 10.1016/j.dib.2018.03.028 (PMC5996234; doi:10.1016/j.dib.2018.03.028)
Supplement: Supplementary file 1 — Supplementary material [file mmc1.docx]

San Sebastian, January 25, 2018

### Declaration

All the authors of the manuscript entitled “Dataset on cellulose nanoparticles from blue agave bagasse and blue agave leaves” (ref. DIB-D-18-00035R1) submitted to Data in Brief, declare that they do not have any conflict of interest.

**
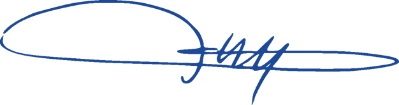
**

Dr. Jalel Labidi

On behalf of the authors
